# Brain-Selective Estrogen Therapy Prevents Androgen Deprivation-Associated Hot Flushes in a Rat Model

**DOI:** 10.3390/ph13060119

**Published:** 2020-06-10

**Authors:** Istvan Merchenthaler, Malcolm Lane, Christina Stennett, Min Zhan, Vien Nguyen, Katalin Prokai-Tatrai, Laszlo Prokai

**Affiliations:** 1 Department of Epidemiology and Public Health, University of Maryland, Baltimore, MD 21201, USA; MLane@som.umaryland.edu (M.L.); christina.stennett@umaryland.edu (C.S.); mzhan@epi.umaryland.edu (M.Z.); 2 Department of Anatomy and Neurobiology, University of Maryland, Baltimore, MD 21201, USA; 3 Department of Pharmacology and Neuroscience, University of North Texas Health Science Center, Fort Worth, TX 76107, USA; Vien.Nguyen@unthsc.edu (V.N.); Katalin.Prokai@unthsc.edu (K.P.-T.); Laszlo.Prokai@unthsc.edu (L.P.); 4 AgyPharma LLC, Mansfield, TX 76063, USA

**Keywords:** prostate cancer, androgen deprivation, brain-selective estrogen prodrug, DHED, male hot flush, rat model, thermoregulation

## Abstract

Hot flushes are best-known for affecting menopausal women, but men who undergo life-saving castration due to androgen-sensitive prostate cancer also suffer from these vasomotor symptoms. Estrogen deficiency in these patients is a direct consequence of androgen deprivation, because estrogens (notably 17β-estradiol, E_2_) are produced from testosterone. Although estrogens alleviate hot flushes in these patients, they also cause adverse systemic side effects. Because only estrogens can provide mitigation of hot flushes on the basis of current clinical practices, there is an unmet need for an effective and safe pharmacotherapeutic intervention that would also greatly enhance patient adherence. To this end, we evaluated treatment of orchidectomized (ORDX) rats with 10β, 17β-dihydroxyestra-1,4-dien-3-one (DHED), a brain-selective bioprecursor prodrug of E_2_. A pilot pharmacokinetic study using oral administration of DHED to these animals revealed the formation of E_2_ in the brain without the appearance of the hormone in the circulation. Therefore, DHED treatment alleviated androgen deprivation-associated hot flushes without peripheral impact in the ORDX rat model. Concomitantly, we showed that DHED-derived E_2_ induced progesterone receptor gene expression in the hypothalamus without stimulating galanin expression in the anterior pituitary, further indicating the lack of systemic estrogen exposure upon oral treatment with DHED.

## 1. Introduction

Hot flushes often negatively impact quality of life because they are associated with acute physical discomfort, as well as sleep disturbances resulting in fatigue, irritability, and forgetfulness [1,2]. Although this most typical climacteric symptom is considered in the context of menopause owing to estrogen deprivation in women, men also flush when they undergo chemical or surgical castration to treat androgen-sensitive prostate cancer or testicular cancer; during the natural, age-related andropause [3,4], or when men with prostate or breast cancer are treated with aromatase inhibitors [5,6,7]. Estrogen deficiency in men is the direct consequence of androgen deprivation, because estrogens, particularly 17β-estradiol (E_2_), are produced from testosterone by enzyme-catalyzed aromatization [8,9]. Among many tissues, the brain in both sexes expresses the aromatase enzyme. Therefore, E_2_ is also produced de novo in the brain [10,11] and, together with E_2_ formed from the peripheral aromatization of testosterone, plays a key role in maintaining normal brain function. Men lacking functional nuclear estrogen receptors (ERs) or aromatase exhibit symptoms similar to menopausal women, including hot flushes [3,12], reinforcing the important role of estrogen in males in this regard.

Estrogens (e.g., transdermal E_2_ patch and the oral synthetic ethinylestradiol (EE)) have been successfully used in men receiving androgen-deprivation therapy (ADT) for the alleviation of hot flushes, cognitive deficits, and depression/anxiety associated with hypoandrogenic conditions [13,14]. However, estrogens in men cause serious side effects such as feminization including gynecomastia and an increased risk of blood clots [3,15]. These are major drawbacks for these patients to utilize the otherwise effective therapy against male climacteric syndromes.

**Figure 1:**
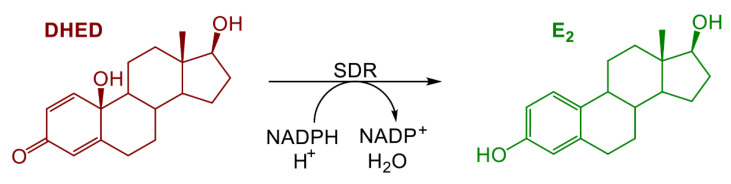
The bioprecursor prodrug 10β, 17β-dihydroxyestra-1,4-dien-3-one (DHED) converts to 17β-estradiol (E_2_) through an enzyme-catalyzed reduction in the brain (SDR: short-chain dehydrogenase/reductase).

To overcome these obstacles, we have developed a CNS-selective estrogen therapy and unequivocally showed the target-selective formation of E_2_ from a unique bioprecursor prodrug 10β,17β-dihydroxyestra-1,4-dien-3-one (DHED), as shown in Figure 1 [16,17,18,19]. Therein, DHED treatment efficiently ameliorated tail skin temperature (TST) elevation in an ovariectomized (OVX) rat model of hot flushes without the increase in circulating serum E_2_ level and associated adverse estrogenic effects in the periphery [16,17]. We hypothesized then that DHED-based estrogen therapy would also be brain-selective in males and, therefore, would prevent ADT-associated hot flushes potentially leading to the development of a safe remedy much needed by hundreds of thousands of men suffering from prostate cancer [20].

Although hot flushes are characteristic events of primates, only a limited number of studies have been published using a handful of non-human primates actually [21,22,23]. Recently, an ewe model was also proposed to study peripheral skin temperature changes associated with menopause [24]. On the other hand, owing to the close association between estrogens and thermoregulation in several species, the use of convenient rodent models has been suitable to evaluate the effects of novel preclinical therapies focused on hot flushes [17,25,26,27]. Since rats dissipate heat through the skin of their tail, measuring changes in TST following ovariectomy is an established model for characterizing this most prominent vasomotor symptom (hot flushes)—especially for evaluating potential pharmacological interventions [17,25]. Morphine-addicted and withdrawn OVX rats have been especially useful due to similarities in symptoms between opioid withdrawal and hot flushes [17,25,26,27]. In the present study, we utilized this model for studying the utility of the orally (p.o.) bioavailable DHED [16,17] on male hot flushes by using orchidectomized (ORDX) rats to mimic the gonadal hormonal status of cancer patients who underwent chemical or surgical castration to implement ADT.

## 2. Results

### 2.1. E_2_ Concentration Increased in the Hypothalamus without Increasing Serum Estrogen after Oral Administration of DHED

**Figure 2:**
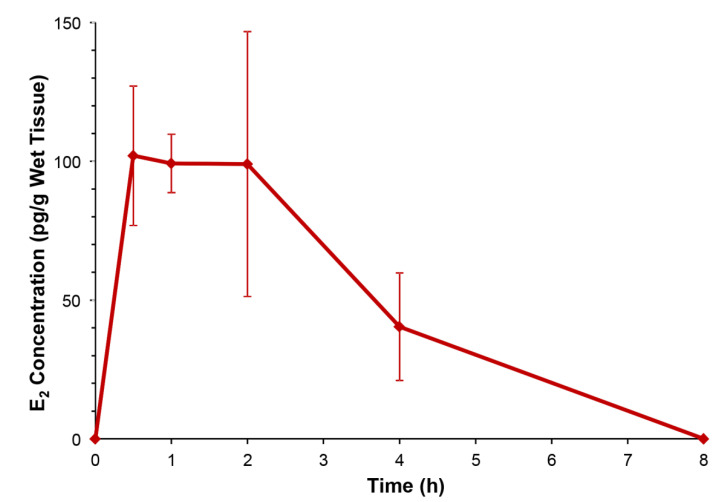
E_2_ concentrations in the hypothalamus of orchidectomized (ORDX) rats after oral (p.o.) administration of DHED (100 µg/kg).

To provide evidence for the DHED-derived E_2_ formation in the ORDX rat brain (Figure 1), we conducted an in vivo experiment relying on the determination of E_2_ content in the hypothalamus through a quantitative assay based on liquid chromatography coupled with tandem mass spectrometry (LC–MS/MS) [28]. This brain region is highly relevant in the present context, as heat-sensitive neurons located in the hypothalamus trigger the descending sympathetic nervous system to dissipate heat through hot flushes [29]. When ORDX animals were treated with a single p.o. dose of DHED (100 µg/kg) and the animals were sacrificed at various time points, E_2_ concentration apparently increased in the hypothalamus, as shown in Figure 2, while our LC–MS/MS assay [28] could not detect any estrogen in this tissue in the untreated control rats. E_2_ reached its maximum value around 30–60 min post drug treatment, which then declined and was no longer quantifiable 8 h after DHED administration.

Importantly, no increase in circulating E_2_ concentrations were detected from serum samples (also collected after the animals were sacrificed) compared to the untreated control levels that were at or below our assay’s ≥10 pg/mL limit of detection. These findings confirmed that DHED administration to ORDX rats also produced central formation of E_2_ without peripheral hormonal liability, similar to OVX rats [16,17,18,19]. In contrast, the clinically used synthetic estrogen (EE, employed here as positive control) produced serum levels reaching a maximum concentration of 840 ± 230 pg/mL EE even after a single 100 µg/kg p.o. dose.

### 2.2. DHED Treatment Blunted Increase of TST in ORDX Male Rats in a Pharmacological Model of Hot Flushes

**Figure 3:**
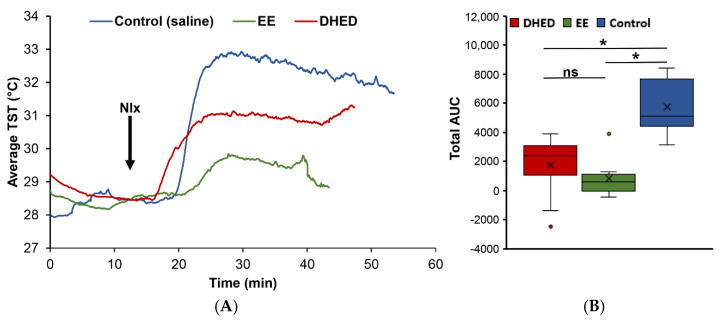
(**A**) Representation of tail skin temperature (TST) changes over time in ORDX animals receiving vehicle (blue), ethinylestradiol (EE; green, 1 mg/kg/day body weight, p.o., twice daily (b.i.d.) for 13 days) and DHED (red, 300 µg/kg/day body weight, p.o., b.i.d. for 13 days) treatments after naloxone (Nlx) challenge. TST rose and reached peak values 10–15 min after treatment with the opioid antagonist, then decreased slowly and returned to baseline (not shown) within an hour and a half. (**B**) Boxplots comparing area under the curve (AUC) distributions by treatment groups specified in panel (**A**). The overall analysis of variance (ANOVA) indicated a statistically significant difference in mean AUC among the three groups. Post hoc Tukey’s multiple comparison tests showed that the mean AUC in the control group was significantly different from the AUCs of the DHED and EE groups (* *p* < 0.05), which were not significantly different (ns) from each other.

Our chosen animal model was validated with the clinically used EE to confirm the desired effect of exogenous estrogen on lowering TST [17,25,26,27] in a male rat pharmacological hot flush model for the first time. In this paradigm, morphine-dependent ORDX animals received twice daily (b.i.d.) drug treatments (1 mg/kg/day p.o. for 13 days; Figure S1), and TST was monitored after naloxone challenge. As shown in Figure 3A, TST began to rise 5 min after the naloxone (Nlx) injection and reached its highest level (approximately 5 °C increase) 15 min after morphine withdrawal in the vehicle-treated control group. EE treatment at this high dose (1 mg/kg body weight), on the other hand, produced an approximate 50% decrease in TST increase at this time point compared to the control group upon Nlx challenge, while an approximate 25% decrease was observed in this measure with the significantly lower dose of prodrug (300 µg/kg body weight, p.o.). Although these changes were less than what we observed by using OVX rats [17,25,26,27] and required a 5 to 10 times higher EE dose to observe attenuation of the effect caused by morphine withdrawal, nevertheless it represented discernible decreases in TST in relation to the no drug treatment. We also found that areas under the TST-time curves (AUCs) were more reliable to assess the effect of the drug treatment than temperature recorded at a somewhat arbitrarily preselected time point such as 15 min after the injection of Nlx. Therefore, AUCs were chosen to evaluate the effect of DHED treatment on the experimental hot flushes in the adapted pharmacological model (Figure 3B).

Accordingly, when animals were treated with 300 µg/kg/day DHED p.o. analogously to that of EE, we measured a statistically significant (*p* < 0.05) decrease in TST in relation to the control in our paradigm. This effect was not significantly different from that of EE treatment at higher concentration (1 mg/kg/day) by the same route and treatment schedule (Figure S1), as shown in Figure 3B. However, the distinguishing feature of DHED is a site-specific E_2_ formation without peripheral hormonal liability, thus without the appearance of E_2_ in the blood (Figure 2).

### 2.3. DHED Treatment Induced Progesterone Receptor (PR) Expression in the Preoptic Area (POA) of the Hypothalamus

**Figure 4:**
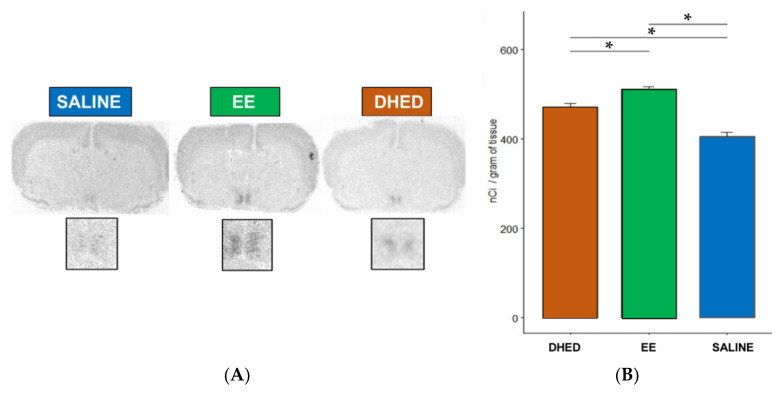
Like the synthetic estrogen EE, DHED stimulated the expression of PR in the preoptic area (POA) after p.o. administration. The optical densities of film exposures over the POA in panel (**A**) of the figure are shown in the chart (**B**). Statistical significance: * *p* < 0.05 by ANOVA followed by post hoc Tukey test.

An independent confirmation of the central formation of E_2_ from DHED in the ORDX rat brain was performed by monitoring PR expression in the hypothalamus. The PR gene is highly sensitive for the presence of E_2_ and is frequently used to evaluate central estrogenic activity of test agents [16,17,30]. As shown in Figure 4, DHED administered p.o. for 13 days b.i.d. increased PR expression in the POA of the hypothalamus, considered the “thermostat“of the body [29], compared to the vehicle-treated group, owing to this prodrug’s conversion to E_2_ in this brain region (Figure 2). Expectedly, the orally administered EE also increased PR expression in the POA of the hypothalamus (Figure 4).

### 2.4. DHED Treatment Did Not Impact Galanin Gene Expression in the Anterior Pituitary (AP)

**Figure 5:**
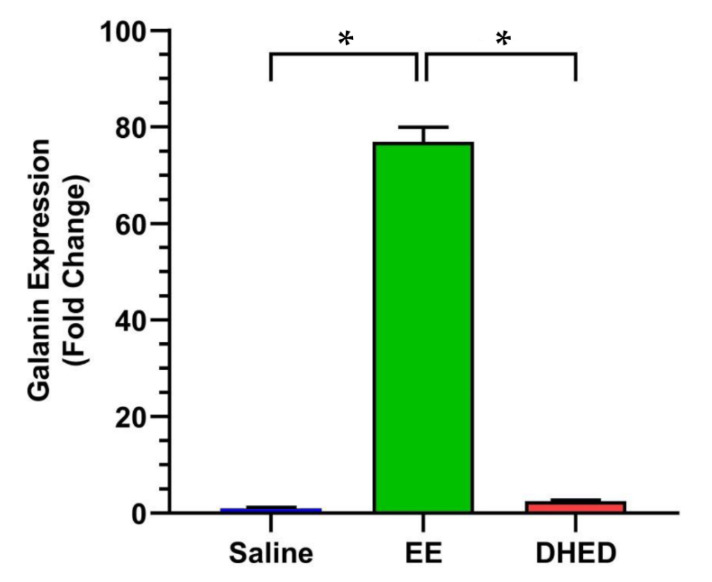
While EE (1 mg/kg/day b.i.d. for 13 days, p.o.) stimulated the expression of galanin in the AP of ORDX rats, DHED (300 µg/kg/day b.i.d. for 13 days, p.o.) had no statistically significant effect compared to saline treatment, indicating lack of its conversion to E_2_ in this gland and the absence of circulating estrogen. Galanin expressions were measured by Singleplex RT-qPCR utilizing a TaqMan probe for rats; * *p* < 0.05 by ANOVA followed by post hoc Tukey–Kramer test.

Galanin is one of the most estrogen-sensitive peripheral genes in the body [16,17,31]. It has been shown that exogenous E_2_ increases its expression on OVX rats by 4000-fold and increases mRNA levels by 50-fold [31]. Therefore, it is important to show that administration of DHED, being inactive as an estrogen [16], would have no effect on this gene expression as the prodrug remains inert in the periphery. Indeed, as shown in Figure 5, DHED treatment did not increase galanin expression measured in the AP with quantitative real-time polymerase chain reaction (qRT-PCR). EE, on the other hand, significantly increased galanin mRNA levels in this organ, confirming the periphery’s exposure to estrogen, as also indicated by our LC–MS/MS bioassay.

## 3. Discussion

Although hot flushes are considered characteristic of perimenopausal and menopausal women, men undergoing ADT due to, e.g., prostate cancer, also suffer from hot flushes, which can be alleviated with estrogen therapy. The adverse effects of such a remedy (most profoundly gynecomastia), however, keep men away from estrogens or discourage adherence to continued therapy among those who initially decide to take it [3,15]. Men suffering from estrogen receptor-positive breast cancer and treated with aromatase inhibitors also exhibit the symptoms characteristic for the menopause discussed above [6,7].

The idea of using estrogen in men looks surprising at first sight, but it has been shown that many actions of estrogen in males are mediated by the aromatization of testosterone to E_2_ [8,9]. About 25–50% of circulating E_2_ is estimated to originate from direct testicular secretion, with the rest resulting from aromatization of testosterone, particularly in adipose tissue, muscle, bone, and brain, where it also may be synthesized de novo [8,12]. Estrogen receptors (ERs), including the classical nuclear ERα, ERβ, and the transmembrane G-protein-coupled ER are expressed throughout the human male reproductive tract and also in the brain [9]. The beneficial role of estrogen in healthy male brain function is supported by the observations that men lacking functional ERs or the aromatase enzyme exhibit symptoms like menopausal women, including hot flushes, depression/anxiety, cognitive impairment, and sleep disturbances [12].

The use of estrogen in men is scientifically sound, but it requires an approach that avoids peripheral side effects. On the basis of our initial preclinical assessment presented here, DHED could fulfill this expectation. As DHED is converted to E_2_ selectively in the brain [16], centrally mediated hypoestrogenic symptoms can be ameliorated without having an adverse impact on the periphery. DHED is a bioprecursor prodrug that is inactive until in vivo metabolism converts it to E_2_ at the target site [16,18,19]. As such, DHED has no affinity to ERs, and its administration also does not stimulate endogenous E2 production by the aromatase pathway [16,19].

We have previously described the pharmacological hot flush model adopted for the purpose of drug discovery and early phase development using OVX rats [16,17,25,27]. To our knowledge, this is, however, the first report involving ORDX rats in this context (Figure 3). Like in OVX animals [16,17], both DHED and EE alleviated TST rises evoked by morphine withdrawal in ORDX rats (Figure 3) owing to DHED-derived E_2_ formation in the hypothalamus (Figure 2)—yet without the exposure of the periphery to the hormone that was also confirmed by the lack of galanin stimulation in the AP (Figure 5). Additionally, PR expression induced in the POA of the hypothalamus [32] further demonstrated DHED’s metabolism into E_2_ at the site of action (Figure 4). 

In conclusion, estrogen therapy with localized action to the brain would be a welcome remedy for neurological symptoms experienced by men undergoing ADT or even a natural andropause. Here, we have shown in a preclinical model that treatment with DHED, the orally bioavailable prodrug of E_2_, fulfills these expectations—it is metabolized to estrogen selectively in the ORDX male brain and, therefore, does not trigger adverse effects in the periphery. This distinguishing feature makes DHED a promising drug candidate for a novel and safe brain-selective estrogen therapy for men.

## 4. Materials and Methods

### 4.1. Chemicals

EE, E_2_, naloxone, and other chemicals were purchased from MilliporeSigma (St. Louis, MO, USA). DHED was synthesized in our laboratory, as described previously [16]. Morphine pellets (containing 75 mg morphine sulfate) were purchased from Murty Pharmaceuticals (Lexington, KY, USA). 17β-Estradiol-13,14,15,16,17,18-^13^C_6_ (^13^C_6_-E_2_) was supplied by Cambridge Isotope Laboratories (Andover, MA, USA).

### 4.2. Animals

All experimental protocols involving animals, including orchidectomy, were approved by the institutional animal care and use committee at the School of Medicine, University of Maryland, Baltimore, MD (approval number: 0217001 on 8 March 2017). Male Sprague-Dawley (SD) rats, 3 months old (≈300 g body weight), were obtained from Charles River Laboratories (Wilmington, DE, USA). The animals were housed in pairs under a 12 h light/dark cycle and given a phytoestrogen-free diet. Water and food were available ad libitum. All animals were cared for according to the National Academy of Sciences guidelines for compassionate use and care of laboratory animals.

### 4.3. Measurement of E_2_ in the Hypothalamus and Serum after Oral Administration of DHED

Drug quantification by LC–MS/MS was based on the principles of isotope dilution [28]. ORDX SD rats (*n* = 5–7) were treated with DHED (100 μg/kg, p.o.) and then euthanized 0.25, 0.5, 1, 2, 4, and 8 h after treatment. The hypothalami were dissected from the harvested brains, followed by tissue homogenization (20% *w*/*v*, pH 7.4 phosphate buffer) and adding ^13^C-labeled internal standard (IS) (^13^C_6_-E_2_, 100 pg) for E_2_ quantitation, as reported previously [16,19,28]. Liquid–liquid extraction was performed with 4 volumes of methyl tert-butyl ether. The organic layers obtained from the extractions were removed and evaporated under a nitrogen stream to yield samples for derivatization and subsequent LC–MS/MS analysis using electrospray ionization. Derivatization of the analyte and the IS was done by dansyl chloride [28]. The dansylated samples were centrifuged at 4500 rpm for 3 min, transferred to autosampler vials, sealed, and assayed using an LC–MS-8050 triple quadrupole tandem mass spectrometer connected to an LC-20AD liquid chromatograph (Shimadzu, Tokyo, Japan). Separation was performed according to our earlier publication using a Phenomenex (Torrance, CA, USA) Kinetex phenyl-hexyl column (50 mm × 2.1 mm i.d., 2.6 µm particles with 100 Ǻ pores), and quantification relied on selected-reaction monitoring [28].

### 4.4. Direct TST Measurements in ORDX Morphine-Dependent Rats

In this morphine-addicted male rat pharmacological hot flush model, we administered saline vehicle as the negative control, EE (1 mg/kg/day) as the positive control, and DHED (300 µg/kg/day), by oral gavage, respectively, b.i.d. Test agents were dissolved in saline vehicle containing 2% (*v*/*v*) dimethyl sulfoxide. The drug treatment protocol of this model is summarized in Figure S1. Accordingly, drug treatment started 5 days after ORDX and continued until the end of the experiments (day 17). On day 11 post-ORDX, we implanted a morphine pellet subcutaneously. On day 13 post-ORDX, we implanted two additional morphine pellets similarly. Three days later, on day 16 post-ORDX, we injected the animals with ketamine (80 mg/kg, intramuscularly), and a thermocouple, connected to a data acquisition system (PhysioTel, Data Sciences International, St. Paul, MN, USA), was taped on the tail approximately 1 inch from the root of the tail to continuously measure TST. Previously, we showed that neither ketamine administration nor the duration of treatment significantly affects neither the temperature rise caused by the morphine withdrawal, nor the suppression of this response by an estrogen [25,27]. Baseline TST was measured for 10 min before the opioid antagonist naloxone (Nlx, 1.0 mg/kg, s.c.) was given to block the effect of morphine. TST was then measured for 40–50 min thereafter. At the end of the experiments, the rats were euthanized, and blood, brain, and pituitaries were collected and processed as reported previously [17].

### 4.5. In Situ Hybridization Histochemistry (ISHH)

At the end of the experiments, animals were euthanized, and brains and APs were removed and immediately frozen on dry ice. The brains were cut with a cryostat, and 20 µm thick sections were placed on gelatin-coated slides. The expression of PR in the POA of the hypothalamus was evaluated with ISHH utilizing radioactive isotope-labeled complementary ribonucleic acid (cRNA) probe complementary to rat PR sequence according to previously published protocol [30]. At the end of the hybridization, we placed slides over X-ray films, and we evaluated and quantified the optical density of the signal over the POA with a C-Imaging (Pittsburg, PA, USA) system.

### 4.6. Quantitative RT-PCR (qRT-PCR) for Galanin Expression in the Anterior Pituitary (AP)

Galanin gene expressions in the AP were measured with qRT-PCR protocol following the procedures reported previously [16,17,32]. Briefly, after mRNA extraction with Trizol reagent (Invitrogen/Thermo Fisher Scientific, Waltham, MA, USA), total RNA was treated with DNase (Invitrogen/Thermo Fisher Scientific, Waltham, MA, USA) according to the manufacturer’s instructions. Then, 500 ng of total RNA per sample was reverse-transcribed into complementary deoxyribonucleic acid (cDNA) in a 20 μL reaction using an iScript cDNA Synthesis Kit (Bio-Rad, Hercules, CA, USA). The procedure was performed using the iQ SYBR Green Supermix (Bio-Rad, Hercules, CA, USA) in a 25 μL reaction using primer pairs for galanin (Fwd 5′-TCTCACCGCTGCTCAAGATG and Rev 5′-GCCATGCTTGTCGCTAAATG) designed with the Accelrys Gene 2.0 software program. The qRT-PCR reaction was run on a MyiQ instrument (Bio-Rad, Hercules, CA, USA). Efficiency and consistency of the cDNA synthesis were determined by amplification of the glyceraldehyde-3-phosphate dehydrogenase (GAPDH) gene as a control. Fold increase was determined by using the 2^−ΔΔC^_T_ method [32].

### 4.7. Statistical Analyses

AUC for each subject was calculated with a trapezoidal method in SAS version 9.4. Because of the unequal group sizes among the experimental (*n* = 12 for DHED treatment and 10 for EE) and control (*n* = 8) groups, we used Tukey’s multiple comparison analyses to compare the distribution of AUC values among the treatment groups, along with ANOVA. For the analysis of treatments on gene expression and E_2_-concentration measurements, we used one- or two-way ANOVA in evaluating simple experiments, followed by appropriate post hoc (Tukey or Tukey–Kramer) tests to compare differences among groups. In all analyses, *p* < 0.05 was considered as statistically significant. Data are displayed as mean ± standard error of the mean (SEM).

## Supplementary Materials

The following are available online at https://www.mdpi.com/1424-8247/13/6/119/s1, Figure S1: Timeline of the hot flush experiments.
