# Supplementary material for: Brain-Selective Estrogen Therapy Prevents Androgen Deprivation-Associated Hot Flushes in a Rat Model
Source: Pharmaceuticals (Basel). 2020 Jun 10;13(6):119. doi: 10.3390/ph13060119 (PMC7344942; doi:10.3390/ph13060119)
Supplement: Supplementary file 1 [file pharmaceuticals-13-00119-s001.pdf]

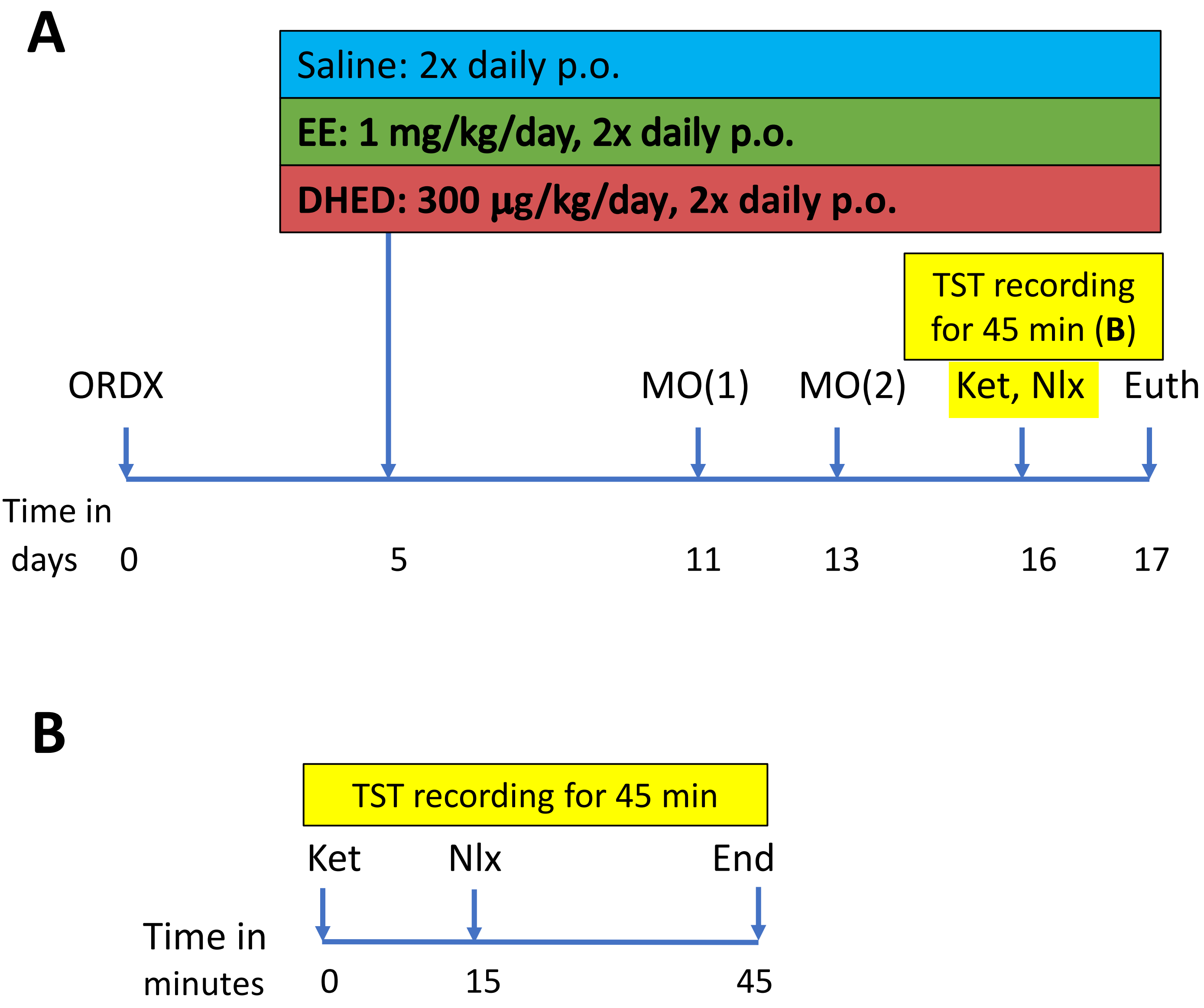


**Figure S1.** Timeline of the hot flush experiments. Male Sprague-Dawley rats were orchidectomized (ORDX) on day 0. Vehicle, EE (1 mg/kg/day) and DHED (300 µg/kg/day) were administered orally, b.i.d., starting on day 5. Treatment with these compounds continued until the end of the experiments (day 14). On day 11 post-ORDX, a morphine (MO) pellet was implanted subcutaneously. On day 13 post-ORDX, two additional morphine pellets were implanted similarly. Three days later (on day16 post-ORDX), the animals were injected with ketamine (Ket, 80 mg/kg, i.m.) and a thermocouple, connected to a data acquisition system (PhysioTel, Data Sciences International, St. Paul, MN), was taped on the tail approximately one inch from the root of the tail. This system allows the continuous measurement of TST. Baseline temperature was measured for 10 min, then naloxone (Nlx, 1.0 mg/kg) was given s.c. (0.2 mL) to block the effect of morphine and TST was measured for 40–50 min thereafter. At the end of the experiments, the rats were euthanized and their brains and pituitaries collected and processed as described in the Materials and Methods section.
